# HIV prevention programmes for female sex workers in Andhra Pradesh, India: outputs, cost and efficiency

**DOI:** 10.1186/1471-2458-5-98

**Published:** 2005-09-24

**Authors:** Lalit Dandona, Pratap Sisodia, SG Prem Kumar, YK Ramesh, A Anod Kumar, M Chalapathi Rao, Elliot Marseille, M Someshwar, Nell Marshall, James G Kahn

**Affiliations:** 1 Health Studies Area, Centre for Human Development, Administrative Staff College of India, Hyderabad, India; 2 Institute for Health Policy Studies and AIDS Research Institute, University of California, San Francisco, USA

## Abstract

**Background:**

Female sex workers and their clients play a prominent role in the HIV epidemic in India. Systematic data on the outputs, cost and efficiency for HIV prevention programmes for female sex workers in India are not readily available to understand programme functioning and guide efficient use of resources.

**Methods:**

Detailed output and cost data for the 2002–2003 fiscal year were obtained using standardised methods at 15 HIV prevention programmes for female sex worker in the state of Andhra Pradesh in southern India. The services provided and their relation to the total and unit economic costs were analysed using regression techniques. The trends for the number of sex workers provided services by the programmes since inception up to fiscal year 2004–2005 were examined.

**Results:**

The 15 programmes provided services to 33941 sex workers in fiscal year 2002–2003 (range 803–6379, median 1970). Of the total number of contacts with sex workers, 41.6% were by peer educators and 58.4% by other programme staff. The number of sex worker contacts in a year by peer educators varied 74-fold across programmes as compared with a 2.7-fold variation in sex worker contacts by other programme staff. The annual economic cost of providing services to a sex worker varied 6-fold between programmes from Indian Rupees (INR) 221.8 (US$ 4.58) to INR 1369 (US$ 28.29) with a median of INR 660.9 (US$ 13.66) and mean of INR 517.8 (US$ 10.70). Personnel salaries made up 34.7% of the total cost, and recurrent goods made up 38.4% of which 82.1% was for condoms. The cost per sex worker provided services had a significant inverse relation with the number of sex workers provided services by a programme (p < 0.001, R^2 ^= 0.75; power function). There was no correlation between the full time equivalents of programme staff and the number of sex workers provided services by the programmes, but there was a modest inverse correlation between the number of sex workers served and the average time spent with each sex worker in the year adjusted for the full-time equivalents of programme staff (p = 0.011, R^2 ^= 0.40; exponential function). The average number of sex workers provided services annually by the first batch of 7 programmes started in early 1999 plateaued after the fourth fiscal year to 3500, whereas the 8 second-batch programmes started in late 2000 reached an average of 2000 sex workers in 2004–2005 with an increasing trend up to this fourth fiscal year.

**Conclusion:**

The HIV prevention efforts in this Indian state would benefit from standardisation of the highly variable services provided by peer educators, who form an important part of the sex worker programmes. The cost per sex worker served decreases with increasing number of sex workers served annually, but this has to be weighed against an associated modest trend of decrease in time spent with each sex worker in some programmes.

## Background

The number of people infected with HIV in India is estimated to be one of the highest in the world [1,2]. The state of Andhra Pradesh in southern India has a population of 80 million, and the sentinel surveillance suggests that it has one of the highest estimated burden of HIV among the Indian states [3]. It has been suggested that female sex workers and their clients play a very prominent role in the HIV epidemic in India, and that effective interventions for them could help substantially curb this epidemic [4]. In the sentinel surveillance of female sex workers at seven locations in Andhra Pradesh in 2004, the HIV prevalence ranged from 8% to 41% with a mean of 16% [5].

HIV prevention programmes for female sex workers form a major component of the HIV prevention efforts in Andhra Pradesh. With recent substantial funding from the Gates Foundation for HIV prevention [6], the number of intervention programmes for female sex workers is increasing rapidly at present in Andhra Pradesh. However, there are few data assessing the outputs, cost and efficiency of the various strategies to control HIV in India [7,8]. Such data are needed for informed planning and efficient utilisation of resources available for HIV control. As part of a study to assess the cost-efficiency of various HIV prevention strategies in Andhra Pradesh, we report data on the outputs, cost and efficiency of HIV prevention programmes for female sex workers.

## Methods

This study forms part of a multi-country effort to study cost and efficiency of HIV prevention in India, Mexico, Russia, South Africa and Uganda by the Prevent AIDS Network for Cost-Effectiveness Analysis (PANCEA) [9]. The methods for the overall multi-country study are described in detail elsewhere [10]. The methods relevant for this report follow.

### Selection of programmes

At the time of starting data collection for this study in mid-2003, 15 HIV prevention programmes for female sex workers were being run by non-governmental organisations in Andhra Pradesh with the support of the Andhra Pradesh State AIDS Control Society. As the HIV epidemic in Andhra Pradesh was initially estimated to be more prevalent in the eastern Coastal region of the state with nine districts, 13 of the 15 programmes were in this region. The other two programmes were in the northern Telangana region of the state that has the state capital and nine other districts, whereas the smaller southern Rayalseema region with four districts had none of these programmes. For this study, we included all the 15 programmes, of which 7 had started functioning in February 1999 and 8 in December 2000.

### Data collection procedures

The initial versions of the data collection instruments from the global PANCEA study were reviewed and refined to suit the context of Andhra Pradesh. The data collection team, consisting of six investigators with background in economics or finance, was involved with the refinement of the instruments and received extensive training to ensure a standardised approach to data collection. A pilot study was done to make final refinements in the data collection format and approach.

Detailed data were initially collected for the April 2002 – March 2003 fiscal year at the 15 sex worker programmes, which included a history of the evolution of the programme, and output and cost data by month. Formal consent of the senior-most person responsible for each programme, generally the director of non-governmental organisation, was obtained to collect data. The programme project director, project manager, accounts officer, outreach coordinator, outreach workers and counsellor were interviewed and the available written records reviewed to obtain the programme data. Each visit started with an interview containing structured open-ended questions on the history of the programme, and operational or community factors that may have affected the programme. Data collection at a programme by three investigators lasted one week. Data were recorded in the field on laptop computers in MS Excel and MS Word files, which after review were entered into an MS Access database.

The number of sex workers provided services annually by each programme since inception up to the 2004–2005 fiscal year was documented in another round of data collection that finished in April 2005.

### Output data

Detailed data were obtained from the written records at the sex worker programmes regarding the services provided by month. The Andhra Pradesh State AIDS Control Society categorises the services delivered by sex worker intervention programmes into four major components: behaviour change communication, sexually transmitted infections care, condom promotion, and creating an enabling environment [11]. Behaviour change communication comprises several types of sessions by outreach workers and peer educators to teach and encourage the sex worker individually and in groups to follow safe sex practices. Sexually transmitted infection care includes the programme staff taking or referring sex workers and occasionally their partners for treatment of sexually transmitted infections (the cost of this is covered by the programme), HIV counselling and referrals to voluntary counselling and testing centres, help in utilising the services of HIV care and support centres, and organising training programmes and sensitisation meets for sexual health service providers such as registered medical practitioners. Condom promotion includes free condom distribution and sale of condoms under social marketing. Creating an enabling environment for sex workers includes meeting with a variety of external stakeholders by organising sensitisation meets with the general public, advocacy meets with the police, media and policy makers, establishing linkages with relevant governmental and non-government organisations, and also assisting sex workers to organise and form community based organisations and with some of their non-sexual health needs. The average time spent by the programme staff with a sex worker for each of the different types of contact was estimated based on information given by programme staff.

### Cost data

The cost of the sex worker programme was divided into five categories: salaries, recurrent goods, recurrent services, office space rentals and capital goods. These cost data were collected for each month, as far as possible. Economic cost was computed, i.e. the true resource cost incurred rather than just the financial cost, as described below.

Salary cost was recorded for all personnel contributing to the work of sex worker programme, which included the project director, project manager, accounts officer, outreach coordinator, outreach workers, counsellor and attender. In addition, the compensation paid by the programme to sex worker peer educators in cash or kind was included in salary costs. Personnel compensation was noted from the official records of the programme. There were no non-salary personnel costs.

The major component of recurrent goods was male condoms. The majority of condoms distributed to the sex workers at these programmes are of a particular brand and are provided free by the Andhra Pradesh State AIDS Control Society. The market price of this brand of condoms is subsidised by 70% by the government. We considered the economic cost of these condoms as what it would have been without the subsidy. Some condoms of other brands are also sold to sex workers under the condom social marketing effort at the cost at which they are procured. Since these condoms are not subsidised, the market price of these condoms was used for economic cost calculations. The other recurrent goods utilised by the sex worker programme included medications for sexually transmitted infections, behaviour change communication materials, stationery and condom outlet boxes. The cost of the other recurrent goods was noted from the official records of the programmes, except for behaviour change communication materials about which information was obtained from the Andhra Pradesh State AIDS Control Society as it supplies these materials to the sex worker programmes.

Recurrent services included local travel, organising various workshops and meets, training for staff and peer educators, computer rentals and maintenance, consultation fees for sexually transmitted infections (investigations were usually not done, diagnosis and treatment were based on symptoms and signs), educational tour for staff, organising special events such as the World AIDS Day and candle light movement, cleaning and building maintenance, telephone, out of station travel, printing, photocopying, electricity, postage and courier, gas and some miscellaneous items. The cost of recurrent services was noted from official records of the programme. The cost for staff training was calculated by including travel fare, per diem, trainer fees, training materials, and training facility cost, information about which was obtained not only from the programme, which incurred some of these costs, but also from the Andhra Pradesh State AIDS Control Society, which incurred many of these costs.

The office of all these programmes run by non-government organisations were located in rented buildings. The details of the monthly rent paid were obtained from the programme records.

Capital goods used for the work of the sex worker programmes included computer and accessories, office furniture, electrical fixtures, telephone, white/black board, scooter, television, bicycle, public address system, video recorder, type writer, stove, refrigerator, audio system, inverter, projector, fax machine, lamination machine, sewing machine, spiral binding machine, water filter, camera, cash box and wall clock. If information about the cost of the capital goods was not available from the sex worker programme or its parent organisation, the market price was determined from retail sellers of these goods. Three quotations for these goods were obtained from the market for the 2002–2003 fiscal year and the average of these taken as the cost. The life of the capital goods was assumed to be five years, and therefore, one-fifth of the cost was allocated to the 2002–2003 fiscal year if the good was used for the full year. If a good was used only for part of the year, the cost was pro-rated for that use. If a capital good, for example a projector or a public address system, was also being used by the parent organisation for work other than that of the sex worker programme, a cost was allocated to the sex worker programme according to the proportion use reported by programme staff.

The average exchange rate of Indian Rupees (INR) 48.40 to a US$ for the 2002–2003 fiscal year was used for INR to US$ conversions [12].

### Quality control

Quality control measures included a thorough pilot study before commencing formal data collection, comprehensive training of a qualified data collection team including their conceptual understanding of all data issues, full back-up and justification for any data recorded, supervision of data collection at each programme by the project coordinator, thorough review by the study team of the data obtained at each programme, and contacting programmes again to obtain information about data issues that needed clarification after the review.

### Data analysis

Data were analysed using SPSS statistical software. The total number of sex workers provided services by each programme, different types of services, services provided per full time equivalent of programme staff, and services provided per sex worker, were analysed. The average economic cost per sex worker served in that fiscal year was taken as the main measure of cost-efficiency, and its relation assessed with the total number of sex workers served. The cost per contact with sex workers was also assessed. Incremental costs for each of the four major types of services provided by the programmes were assessed using a multiple regression model. Statistical significance was defined as p < 0.05.

The relation between the number of sex workers provided services and the intensity of services in the form of time spent with each sex worker was assessed. Comparison was done between the services provided by programmes that were started in the first phase in February 1999 (first batch) and those started later in December 2000 (second batch).

## Results

A total of 33941 female sex workers were provided services by the 15 programmes during the 2002–2003 fiscal year. The managers for these programmes estimated that there were a total of 42900 female sex workers in the areas covered by these programmes in this fiscal year. The number of sex workers provided services annually by each programme ranged from 803 to 6379, with a median of 1970 and mean of 2263 (Table 1). Based on estimates of the types of female sex workers served by each programme, of the total sex workers served by all the programmes considered together 57% were street-based, 27% brothel-based and 16% home-based. There were a total of 245367 contacts with sex workers. The average number of contacts of programme staff with each sex worker in a year ranged 6.5-fold from 2.6 to 16.9 with a median of 8 for the different programmes (Table 1). There were a total of 49142 contacts with external stakeholders. The average number of contacts of programme staff other than peer educators with the external stakeholders in a year ranged 15-fold from 98 to 1463 with a median of 215 (Table 1).

**Table 1:** Number and intensity of services provided by the sex worker programmes in fiscal year 2002–2003.

Programme number*	Number of sex workers provided services	Total number of contacts with sex workers	Number of contacts with each sex worker	Total full time equivalents of staff	Number of contacts with sex workers per staff full time equivalent	Total number of contacts with all types of external stakeholders†	Total full time equivalents of staff other than peer educators	Number of contacts with external stakeholders per staff full time equivalent other than peer educators‡
8	6379	57077	8.9	26.00	2196	1002	8.50	118
10	4690	12477	2.7	18.75	665	1989	10.00	199
5	3847	9953	2.6	20.04	497	2880	9.79	294
9	2552	26976	10.6	15.66	1722	14869	10.16	1463
7	2336	18920	8.1	26.91	703	2015	11.66	173
11	2156	18358	8.5	14.35	1279	1111	6.85	162
1	2102	10833	5.2	14.78	733	1320	10.03	132
12	1970	14327	7.3	19.91	719	8244	10.41	792
15	1442	13064	9.1	20.33	643	1793	8.33	215
4	1350	22766	16.9	17.00	1339	2636	8.25	320
13	1274	9043	7.1	28.83	314	3016	10.33	292
2	1229	8867	7.2	11.58	766	816	8.33	98
6	915	10568	11.5	18.33	577	3433	8.33	412
14	896	7207	8.0	26.08	276	3248	8.33	390
3	803	4931	6.1	15.25	323	770	7.75	99
Total	33941	245367	7.2	293.80	835	49142	137.05	359

* Programmes arranged in decreasing order of the number of sex workers to whom they provided services in fiscal year 2002–2003.

† External stakeholders include general public, police, media, policy makers, and governmental and non-governmental agencies.

‡ Peer educators excluded here as contact with external stakeholders is made by other programme staff.

Of the total number of contacts with sex workers by the 15 programmes, 58.4% were by staff other than peer educators and 41.6% by peer educators. The full time equivalents of peer educators with the different programmes had a wider range of 3.3 to 18.5 (median 9.5) as compared with the range of 6.9 to 11.7 (median 8.5) for the programme staff other than peer educators (Table 2). In addition, the number of sex worker contacts in a year per full time equivalent of peer educators ranged 74-fold from 36 to 2626 (median 259) whereas those per full time equivalent of programme staff other than peer educators ranged 2.7-fold from 568 to 1520 (median 958) (Table 2). There was no significant correlation between the number of full time equivalents of peer educators in a programme and the number of sex worker contacts per peer educator (p = 0.66, R^2 ^= 0.01; linear function), or between full time equivalents of programme staff other than peer educators in a programme and the number of sex worker contacts per staff (p = 0.26, R^2 ^= 0.09; linear function), or between sex worker contacts per full time equivalents of programme staff other than peer educators in a programme and sex worker contacts per full time equivalents of peer educators (p = 0.20, R^2 ^= 0.12; linear function).

**Table 2:** Intensity of services provided by peer educators and other programme staff in fiscal year 2002–2003.

Programme number*	Number of sex workers provided services	Full time equivalents of staff other than peer educators								Total number of contacts with sex workers other than peer educators	Contacts with sex workers per staff other than peer educators	Full time equivalents of peer educators	Total number of contacts with sex workers by peer educators	Contacts with sex workers per peer educator
							
		Project director	Project manager	Accounts officer	Outreach coordinator	Outreach worker	Counsellor	Office assistant	Total					
8	6379	0.33	1.08	1.00	1.00	4.00	1.00	0.08	8.50	11114	1308	17.50	45963	2626
9	2552	0.33	1.00	1.00	0.92	4.00	1.00	1.92	10.16	15332	1509	5.50	11644	2117
4	1350	0.33	1.00	1.00	0.00	4.00	0.92	1.00	8.25	8426	1021	8.75	14340	1639
11	2156	0.27	0.75	0.58	0.00	3.34	1.08	0.83	6.85	7503	1095	7.50	10855	1447
2	1229	0.33	1.00	1.00	0.00	4.00	1.00	1.00	8.33	5507	661	3.25	3360	1034
12	1970	0.33	1.00	1.00	1.00	5.08	1.00	1.00	10.41	9581	920	9.50	4746	500
6	915	0.33	1.00	1.00	0.00	4.00	1.00	1.00	8.33	7918	951	10.00	2650	265
1	2102	1.00	1.00	1.03	1.00	4.00	1.00	1.00	10.03	9605	958	4.75	1228	259
13	1274	0.33	1.00	1.00	0.00	6.00	1.00	1.00	10.33	6830	661	18.50	2213	120
5	3847	0.33	1.04	1.00	1.00	4.25	1.17	1.00	9.79	8901	909	10.25	1052	103
15	1442	0.33	1.00	1.00	0.00	4.00	1.00	1.00	8.33	11960	1436	12.00	1104	92
7	2336	0.33	1.00	1.00	1.00	6.33	1.00	1.00	11.66	17728	1520	15.25	1192	78
3	803	0.33	1.00	1.00	0.00	3.75	0.67	1.00	7.75	4405	568	7.50	526	70
10	4690	1.00	1.00	1.00	1.00	4.00	1.00	1.00	10.00	11996	1200	8.75	481	55
14	896	0.33	1.00	1.00	0.00	4.00	1.00	1.00	8.33	6574	789	17.75	633	36
Total	33941	6.23	14.87	14.61	6.91	64.75	14.84	14.83	137.05	143380	1046	156.75	101987	651

* Programmes arranged in decreasing order of the number of contacts with sex workers per peer educator in fiscal year 2002–2003.

The major portion of the contacts with sex workers by programme staff was related to behaviour change communication (93.7%) (Table 3). The number of such contacts per sex worker in a year ranged from 2 to 16.6 (median 7.4) for the different programmes. The number of sexually transmitted infection care related contacts in a year ranged from 0.15 to 0.84 (median 0.39) per sex worker provided services by the different programmes (Table 3). Of these contacts related to sexually transmitted infections, 77.9% were for referral of sex workers for treatment of sexually transmitted infections, 14.9% for HIV counselling, referral to voluntary counselling and testing centres, and referral to care and support centres, 5.5% for training of sexual health service providers, and 1.7% for referral of partners of sex workers for treatment of sexually transmitted infections. The number of condoms distributed by the different programmes in a year per sex worker provided services ranged from 46 to 432 with a median of 168 (Table 3). Of all the condoms distributed, 95% were given free and 5% sold as part of the condom social marketing effort.

**Table 3:** Components of services provided by sex worker programmes in fiscal year 2002–2003.

Programme number*	Number of sex workers provided services	Behaviour change communication contacts	Behaviour change communication contacts per sex worker	Sexually transmitted infection care related contacts	Sexually transmitted infection care related contacts per sex worker	Condoms distributed	Condoms distributed per sex worker	Contacts for creating enabling environment	Contacts for creating enabling environment per sex worker
8	6379	56141	8.8	987	0.15	590922	93	951	0.15
10	4690	10688	2.3	1326	0.28	215861	46	2452	0.52
5	3847	7556	2.0	746	0.19	419583	109	4531	1.18
9	2552	25400	10.0	2045	0.80	356658	140	14400	5.64
7	2336	18221	7.8	1449	0.62	393102	168	1265	0.54
11	2156	18173	8.4	347	0.16	140285	65	949	0.44
1	2102	6671	3.2	1222	0.58	554146	264	4260	2.03
12	1970	14155	7.2	509	0.26	448004	227	7907	4.01
15	1442	12074	8.4	1214	0.84	448704	311	1569	1.09
4	1350	22409	16.6	369	0.27	173858	129	2624	1.94
13	1274	8647	6.8	654	0.51	300750	236	2758	2.16
2	1229	8458	6.9	485	0.39	165670	135	740	0.60
6	915	10316	11.3	292	0.32	179390	196	3393	3.71
14	896	6600	7.4	655	0.73	387237	432	3200	3.57
3	803	4498	5.6	443	0.55	138490	172	760	0.95
Total	33941	230007	6.8	12743	0.38	4912660	145	51759	1.52

* Programmes arranged in decreasing order of the number of sex workers to whom they provided services in fiscal year 2002–2003.

The number of contacts by the programmes related to creating an enabling environment for sex workers ranged from 0.15 to 5.64 (median 1.18) per sex worker provided services (Table 3). Of these contacts, 77% were with the community at large in the form of mass or special events, 12.1% were with other external stakeholders in the form of advocacy meets and with agencies to enhance linkages, and 10.9% were with sex workers regarding formation of community based organisations and facilitation of non-sexual health needs. There was no significant correlation between behaviour change communication contacts per sex worker by a programme on the one hand and sexually transmitted infections related contacts per sex worker provided services (p = 0.98, R^2 ^= 0.00; linear function) or number of condoms distributed per sex worker provided services (p = 0.95, R^2 ^= 0.00; linear function) or number of contacts related to creating an enabling environment for sex workers (p = 0.30, R^2 ^= 0.08; linear function) on the other hand.

The total economic cost of services provided by the 15 programmes during the 2002–2003 fiscal year was INR 17,575,858 (US$ 363,138), of which personnel cost made up 34.7%, recurrent goods 38.4% (82.1% of this was for condoms and 12.6% for medicines for sexually transmitted infections), recurrent services 21.1% (the largest component was local travel, which made up 23.2% of this), rentals 4.1%, and capital goods 1.7%. There were modest variations in these proportional costs among some of the programmes (Table 4). The total financial cost was 20.8% less than the economic cost due to the subsidy on condoms.

**Table 4:** Cost and efficiency of sex worker programmes in fiscal year 2002–2003.

Programme number*	Number of sex workers provided services	Total number of contacts with sex workers	Total economic cost		Percent of economic cost					Cost per sex worker provided services		Cost per sex worker contact	
			
			INR	US$	Personnel	Recurrent goods	Recurrent services	Rentals	Capital goods	INR	US$	INR	US$
10	4690	12477	1040254	21493	46.9	19.6	24.3	5.8	3.5	221.8	4.58	83.4	1.72
8	6379	57077	1969785	40698	34.1	41.4	20.5	2.8	1.2	308.8	6.38	34.5	0.71
11	2156	18358	725710	14994	36.6	27.4	28.1	5.8	2.1	336.6	6.95	39.5	0.82
5	3847	9953	1327582	27429	33.1	37.6	22.0	4.5	2.7	345.1	7.13	133.4	2.76
9	2552	26976	1482412	30628	32.2	39.0	23.6	3.2	2.0	580.9	12.00	55.0	1.14
12	1970	14327	1278085	26407	35.9	45.0	14.3	3.6	1.1	648.8	13.40	89.2	1.84
7	2336	18920	1526201	31533	32.4	37.4	24.0	3.9	2.3	653.3	13.50	80.7	1.67
2	1229	8867	812295	16783	45.4	30.3	18.9	4.9	0.6	660.9	13.66	91.6	1.89
4	1350	22766	930607	19228	36.2	32.3	26.9	3.9	0.8	689.3	14.24	40.9	0.84
15	1442	13064	1065021	22005	27.3	52.3	14.8	4.5	1.1	738.6	15.26	81.5	1.68
1	2102	10833	1605711	33176	29.2	52.0	13.3	4.1	1.4	763.9	15.78	148.2	3.06
3	803	4931	673355	13912	45.4	24.3	24.2	4.9	1.1	838.5	17.33	136.6	2.82
13	1274	9043	1089778	22516	34.5	33.9	26.2	3.9	1.5	855.4	17.67	120.5	2.49
6	915	10568	822442	16993	38.7	36.8	18.2	4.4	1.9	898.8	18.57	77.8	1.61
14	896	7207	1226620	25343	27.6	44.5	22.6	3.8	1.4	1369.0	28.29	170.2	3.52
Total	33941	245367	17575858	363138	34.7	38.4	21.1	4.1	1.7	517.8	10.70	71.6	1.48

* Programmes arranged in increasing order of cost per sex worker provided services in fiscal year 2002–2003.

**Figure 1:**
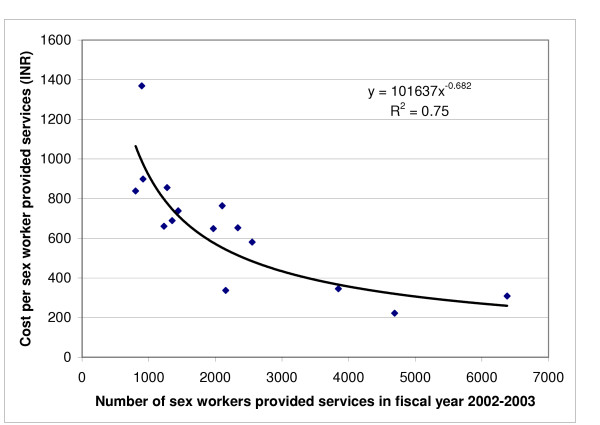
Relation between the number of sex workers provided services in fiscal year 2002–2003 by the 15 programmes and the cost per sex worker (p < 0.001). INR is Indian Rupee (1 US$ = INR 48.40 in 2002–2003).

**Figure 2:**
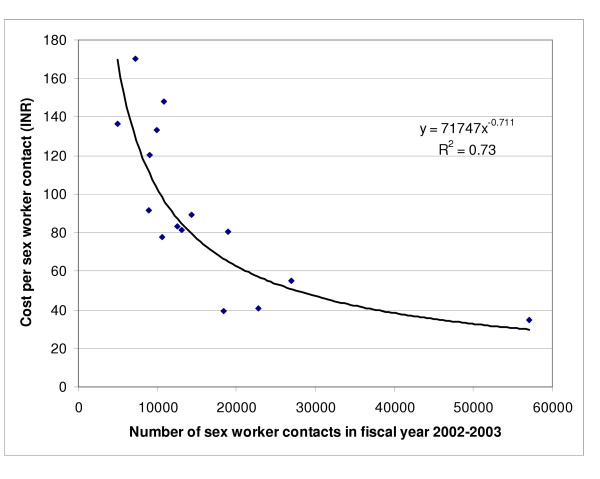
Relation between the number of sex worker contacts in fiscal year 2002–2003 by the 15 programmes and the cost per contact (p < 0.001). INR is Indian Rupee (1 US$ = INR 48.40 in 2002–2003).

The economic cost for each sex worker provided services varied 6-fold between the 15 programmes from INR 221.8 (US$ 4.58) to INR 1369 (US$ 28.29), with a median of INR 660.9 (US$ 13.66) (Table 4). The average cost of providing services to each sex worker at all the 15 programmes combined was INR 517.8 (US$ 10.70). The economic cost for each sex worker contact varied 5-fold between the 15 programmes from INR 34.5 (US$ 0.71) to INR 170.2 (US$ 3.52), with a median of INR 83.4 (US$ 1.72) (Table 4). The average cost of each sex worker contact at all the 15 programmes combined was INR 71.6 (US$ 1.48). Both, the cost per sex worker provided services and the cost per sex worker contact for the different programmes, were significantly inversely associated with scale. The best fit for this relation was obtained with the power function (Figures 1 and 2), with which scale explained 75% of the variability in cost per sex worker provided services and 73% of the variability in cost per sex worker contact by programme staff. There was also a statistically significant direct linear relation between the cost of providing services to each sex worker and the cost of each sex worker contact at a programme, and this explained 35% of the variability (p = 0.02, R^2 ^= 0.35).

**Figure 3:**
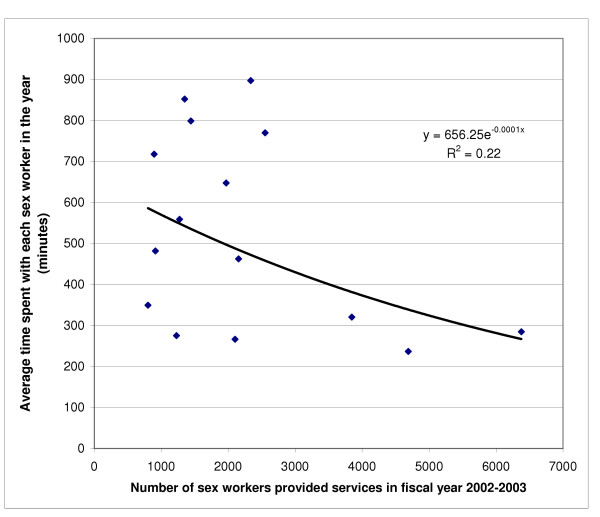
Relation between the number of sex workers provided services in fiscal year 2002–2003 by the 15 programmes and the average time spent with each sex worker in the year (p = 0.079).

**Figure 4:**
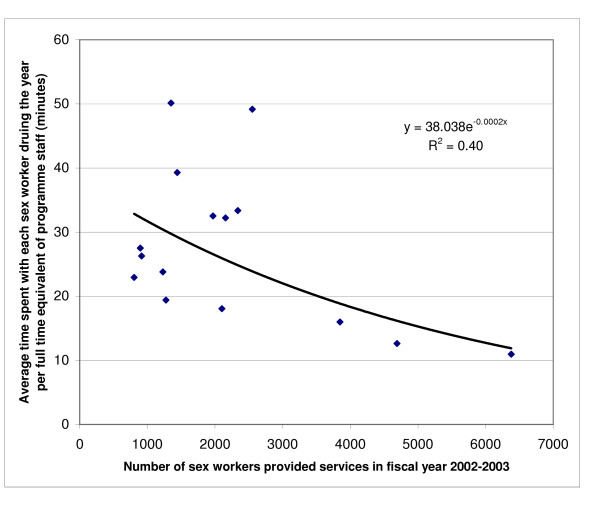
Relation between the number of sex workers provided services in fiscal year 2002–2003 by the 15 programmes and the average time spent with each sex worker in the year adjusted for the full time equivalents of programme staff (p = 0.011).

There was no significant correlation between the full time equivalents of programme staff and the number of sex workers provided services by the programmes (p = 0.42, R^2 ^= 0.05; linear function). Not only was there no inverse relation between the number of sex workers provided services and the contacts per sex worker by the programmes, there was a modest positive linear relation (p = 0.02, R^2 ^= 0.34). However, there was also a borderline inverse correlation between the number of sex workers served and the average time spent with each sex worker by the programme staff during the year (p = 0.08, R^2 ^= 0.22; exponential function) (Figure 3), which became statistically significant after adjusting for the number of full time equivalents of staff in each programme (p = 0.01, R^2 ^= 0.40) (Figure 4).

The 15 programmes had started in two phases, 7 in February 1999 (first batch) and 8 in December 2000 (second batch), resulting in a difference of a 1.75 years in their duration. The first batch programmes were started in places estimated to have the highest numbers of sex workers in the state, followed by the second batch programmes at places also estimated to have sizeable number of sex workers. All 7 first batch programmes (numbers 8, 10, 5, 9, 7, 1, 12 in Table 1) fell among the 8 programmes that served the highest number of sex workers in the 2002–2003 fiscal year. Of the 7 programmes with the least cost per sex worker served in this fiscal year, 6 were first batch programme (numbers 10, 8, 5, 9, 12, 7 in Table 4).

**Figure 5:**
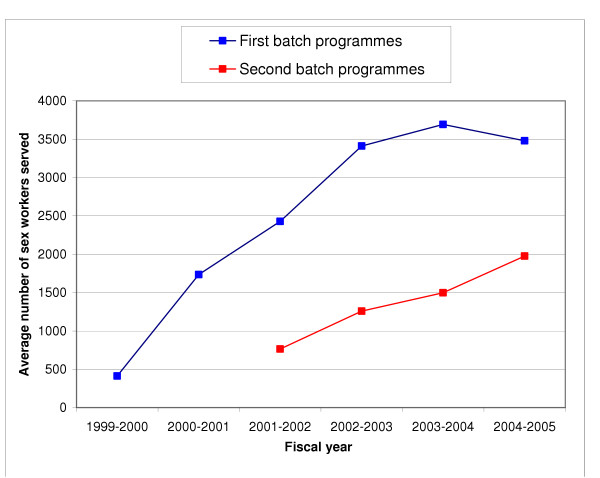
Changes in the average number of sex workers provided services annually by each of the first and second batch programmes since inception.

Since scale was a major determinant of efficiency, we studied the trends of the average number of sex workers provided services annually by the first and second batch programmes since inception (Figure 5). The fiscal year 1999–2000 was mostly spent in planning and establishing the first batch of sex worker programmes in Andhra Pradesh. The average number of sex workers served by each of the first batch programmes increased over the first four fiscal years and then settled around 3500 annually. The second batch of programmes, established at places estimated to have the next highest number of sex workers after the places where the first batch was started, has now finished the first four fiscal years over which it has shown an increasing trend so far that has reached an annual average of about 2000 sex workers per programme served in fiscal year 2004–2005.

Considering the 2002–2003 fiscal year data from each programme as a data point, multiple regression was used to assess the relationship between total economic cost and the four major functions of these programmes, i.e. behaviour change communication contacts with sex workers, condoms distributed, sexually transmitted infections related contacts, and contacts to enhance enabling environment, which revealed the following relation:

 = 372887 + 8.10 P + 1.67 Q + 136.78 R + 3.64 S

where Ĉ is the total economic cost in INR, P is the number of behaviour change communication contacts with sex workers, Q is the number of condoms distributed, R is the number of sexually transmitted infections related contacts, and S is the number of contacts to enhance enabling environment. This model explained 88% of the variability in the economic cost (R^2 ^= 0.88, standard error = 125362) and the fit was significant at p < 0.001. In this model, the constant was significant at p = 0.001 (F = 27.38; degrees of freedom: 4 for regression, 10 for residual, 14 total). Two of the predictor variables were statistically significant (number of behaviour change communication contacts with sex workers and number of condoms distributed) whereas the other two predictor variables were not (number of sexually transmitted infections related contacts and number of contacts to enhance enabling environment at) (Table 5). This model suggests that apart from the constant cost of INR 372887 (US$ 7704) for a programme, the additional cost for each behavioural change communication contact was INR 8.10 (US$ 0.17), for each condom distributed INR 1.67 (US$ 0.03), for each sexually transmitted infections related contact INR 137 (US$ 2.83), and for each contact to enhance enabling environment INR 3.64 (US$ 0.08).

**Table 5:** Coefficients in the multiple regression model and their significance.

Variable	Coefficient (βi)	Standard error	t	Significance
Constant	372887.07	83932.49	4.443	0.001
Number of behaviour change communication contacts with sex workers	8.10	2.84	2.852	0.017
Number of condoms distributed	1.67	0.27	6.298	0.000
Number of sexually transmitted infections related contacts	136.78	85.31	1.603	0.140
Number of contacts to enhance enabling environment	3.64	10.91	0.334	0.745

Dependent variable: Total economic cost

Applying these incremental costs to the total services provided by the 15 programmes (Table 3), of the total economic cost in 2002–2003, 68.3% was variable. Of the variable cost, 15.5% was for behavioural change communication, 14.5% for sexually transmitted infections, 68.4% for condoms, and 1.6% for enhancing enabling environment.

## Discussion

In this study of 15 HIV prevention programmes for female sex workers in the Indian state of Andhra Pradesh, the most important determinant of cost per sex worker provided services was scale. As the number of sex workers provided services increased, the cost per sex worker decreased. This was observed for the entire range of scale of the 15 programmes – 803 to 6373 sex workers provided services in the 2002–2003 fiscal year. However, this should be interpreted with certain caveats. There was a trend towards a modest decrease in the average time spent with each sex worker as the total number of sex workers served increased. The distribution of this relationship (Figure 4) implies that at least for some low-to-medium scale programmes it should be possible to increase the efficiency without compromising time spent with each sex worker. Interestingly, there was also a modest increase in the number of contacts with each sex worker as the total number of sex workers served increased. However, no standardised estimates are available about how many contacts and how much time with each sex worker would be optimal for providing effective HIV prevention services in India. Our data imply that although the efficiency increases with the scale of the programme, further development of sex worker programmes should also include assessment of the optimal number of contacts and time spent with sex workers for the various types of services provided in order to make these programmes not only efficient but also effective. This would have to include variables such as geographical coverage of the programme and transport availability, relative mix of fresh and old sex workers covered by the programme, and relative mix of street-based, home-based and brothel-based sex workers covered.

The first batch of 7 programmes started in early 1999 had reached an average scale of about 3500 sex workers per year per programme by the fourth fiscal year since inception and this was maintained for three fiscal years so far. The second batch of 8 programmes started in late 2000 showed an increasing trend in scale over the four fiscal years so far since inception, with an average of about 2000 sex workers served per year per programme. Since the second batch of programmes were started in places that had on average somewhat fewer sex workers than the first batch of programmes, it is possible that the second batch may not reach the average scale reached by the first batch. There was an increase of 57% for the average scale from 1258 to 1976 from fiscal year 2002–2003 to 2004–2005 for the second batch programmes. The average cost per sex worker provided services by the second batch programmes in 2002–2003 was INR 730 (US$ 15.08). If the equation in Figure 1 for the relation of scale with efficiency were applied, the cost per sex worker served by the second batch programmes in 2004–2005 would have decreased by 21% to INR 576 (US$ 11.90) at the 2002–2003 constant INR/US$. On the other hand, the average cost per sex worker provided services by the first batch programmes in 2002–2003 was INR 428 (US$ 8.85), which would have likely remained stable up to 2004–2005 as these programmes had a stable average scale of about 3500 per year over this period.

Another study of 17 sex worker programmes, 9 in Andhra Pradesh and 8 in Tamil Nadu – another state in southern India, found a U-shaped relationship between scale and efficiency for the 2001–2002 fiscal year with the cost per sex worker being lowest in the range of 1000–1700 sex workers served per year by a programme and then rising [13]. This study reported a median cost of US$ 19.20 (range 9.86–50.70) per sex worker served for a range of 200 to 2008 sex workers served per year by the 17 programmes. Direct comparison of this study with ours may not be possible because of methodological differences, such as the different scale of programmes in the two studies. Our study included much larger programmes; the range of sex workers served per year in the 2002–2003 fiscal year was 803 to 6373, with 7 of the 15 programmes having served over 2000 sex workers and 3 programmes substantially above this number (3847, 4690 and 6379). We did not find a U-shaped relation between scale and efficiency in our data, as the efficiency continued to improve with scale, though the rate of improvement decreased with increasing scale. The values for the cost per sex worker served in our data (median US$ 13.66, range US$ 4.58–28.29) were also lower than in the above study, again likely due to the higher scale in our study. The relationship between scale and efficiency of sex worker programmes is more likely to be revealed if the range of scales of programmes studied is wide. For our study, we included all the 15 HIV prevention programmes for female sex workers that were being run in the fiscal year 2002–2003 by non-governmental organisations in Andhra Pradesh with the support of the Andhra Pradesh State AIDS Control Society.

Peer educators, sex workers who are trained to deliver HIV prevention services to other sex workers, are an important component of HIV prevention programmes for female sex workers in this Indian state with 41.6% of all contacts with sex workers in the fiscal year 2002–2003 done by peer educators for the 15 programmes studied. As the peer educators are themselves sex workers, they are likely to have good access to and rapport with other sex workers. There was a very high 74-fold variation between the programmes for the number of sex worker contacts in a year per peer educator, whereas the variation for the number of sex worker contacts per programme staff other than peer educators was much smaller (2.7-fold). If attempts were made to standardise the output of peer educators, the overall output and efficiency of sex worker programmes in this Indian state would improve. It should be noted though that our study did not assess effectiveness, which would also have to receive attention in attempts to increase efficiency.

The contact by programme staff with sex workers for behavioural change communication forms a major portion of the work of the programme (Table 3). This activity also overlaps with condom distribution. Though the number of contacts for behavioural change communication for all programmes considered together was 18 times higher than the contacts related to sexually transmitted infection care in 2002–2003, the total variable costs for these two activities were similar as the cost of each behaviour change contact (INR 8.10, US$ 0.17) was 17 times lower than the cost of each sexually transmitted infection care related contact (INR 137, US$ 2.83). Based on the multiple regression model for economic cost, apart from the fixed cost needed to run the programmes (31.7% of the total economic cost), of the variable cost two-third was accounted for by the 4.91 million condoms distributed by the 15 programmes in 2002–2003. This suggests that availability of low-cost condoms of reasonable quality is very important for increasing efficiency of sex worker programmes.

The estimates of outputs, cost and efficiency of HIV prevention services in India are generally only scantily available [7,8]. The outputs, cost and efficiency estimates, relationship of efficiency with scale, and the unit incremental costs for each of the major activities of the sex worker programmes, presented in this paper could be useful for planning sex worker programmes and estimating the resources needed by them in Andhra Pradesh and other states in India. This is particularly relevant as there has been a major recent increase in the number of sex worker programmes in Andhra Pradesh and a few other Indian states with substantial funding from the Gates Foundation [6]. The data presented in this paper also highlight that the role of peer educators needs to be standardised and that the relationship between efficiency and effectiveness needs to be understood further.

This study is part of the multi-country PANCEA study [9,10]. The outputs and cost-efficiency estimates of HIV prevention programmes for sex workers from India can be compared with similar estimates from other countries in the PANCEA study using similar methodology. Policy-relevant comparisons of these estimates would also be possible with other HIV prevention strategies in Andhra Pradesh that we have undertaken with similar methodology. For example, we have recently reported cost-efficiency estimates for HIV voluntary counselling and testing in Andhra Pradesh [14]. Standardised comparisons of cost-efficiency estimates for HIV prevention services are needed across countries and across the different types of prevention in the background of the considerable attention recently to estimating the resources needed for HIV control, which would enable more firm local and global estimates for planning.

## Conclusion

The annual economic cost of providing services to a sex worker varied 6-fold between the 15 HIV prevention programmes for female sex workers that were being run in the fiscal year 2002–2003 by non-governmental organisations in the southern Indian state of Andhra Pradesh. The cost per sex worker served decreased with increasing number of sex workers served annually by a programme, and this was associated with a modest trend of decrease in time spent with each sex worker in some programmes. However, it should be possible for at least some low-to-medium scale programmes to increase scale and efficiency without compromising time spent with each sex worker. The services provided by peer educators, who form an important part of the sex worker programmes, were highly variable, standardisation of which would be useful. The data of this study imply that although the efficiency increases with the scale of the programme, further development of sex worker programmes should also include assessment of the optimal number of contacts and time spent with sex workers for the various types of services provided in order to make these programmes not only efficient but also effective.

## Competing interests

The author(s) declare that they have no competing interests.

## Authors' contributions

LD led the PANCEA study in India, guided the design, data collection and analysis, and wrote the initial draft of this paper. PS contributed to the design, data collection and analysis. SGPK, YKR, AAK, MCR and SM contributed to data collection and analysis. NM participated in the design of data collection instruments and the review of collected data. EM and JGK oversaw the PANCEA design and contributed to the analytical design and presentation. All authors read and approved the final manuscript.

## Pre-publication history

The pre-publication history for this paper can be accessed here:


